# Rome, a Policy without Politics: The Participatory Process for a Metropolitan Scale Food Policy

**DOI:** 10.3390/ijerph17020479

**Published:** 2020-01-11

**Authors:** Giampiero Mazzocchi, Davide Marino

**Affiliations:** Department of Biosciences and Territory, University of Molise, 86100 Campobasso, Italy; dmarino@unimol.it

**Keywords:** food policy, food system sustainability, urban agriculture, local development, food governance, stakeholder engagement

## Abstract

In light of the challenges that all cities face today, food is offered as a prism through which to read and intervene on various areas that affect the quality of life of the population: circular economy, urban metabolism, social relations, economies, and food quality. In the Roman context, in recent years, numerous initiatives have revitalized the debate on food and brought the discussion to the center of the interest of an ever-increasing number of citizens. However, these experiences appear unrelated and there is a lack of coordination and political coherence. Faced with this evidence, starting from a territorial analysis, this contribution analyzes the process that led a local group of stakeholders to formulate a proposal for a food policy for the city of Rome. The proposal contains a series of possible actions that aim, on the one hand, to recompose the relations between the city and its territory, with a view to re-localization and re-territorialization of agro-food productions and, on the other hand, to reconnect the economic and social relations that the industrialization of food chains has compromised. The network analysis of the bottom-up process, which mainly investigates networking and negotiation skills between various interests, is carried out and related to a careful analysis of the food system in the Roman context. Furthermore, an overview of the state of the art of urban food policies in Italy has been provided to better contextualize the study case. The findings show actors and topics involved in the process, identifying further development towards a more comprehensive participatory process for a systemic food strategy at the metropolitan level.

## 1. Introduction: Why Cities Need Food Policies

The recent report of the Lancet Commission “Food in the Anthropocene” [1] confirms some dramatic evidence concerning today’s food systems: (1) The current composition of diets does not allow adequately feed the entire world population preserving, at the same time, ecosystems and natural resources. (2) Food consumption patterns of richer economies contribute substantially to global warming, producing huge amounts of greenhouse gases, while those that are emerging in developing countries are based on increasing consumption of red meat [2] and cereals, exacerbating pressures on water resources, soil, and biodiversity [2,3,4] (In the period 1961–2016, the greenhouse gas emissions due to agricultural production increased from 3.1 Gt CO_2_-eqyr-1 to 5.8 Gt 17 CO_2_-eqyr-1, mainly due to breeding, the use of synthetic fertilizers, and the cultivation of rice [2]). (3) Food production, processing, and distribution are among the most impacting factors in terms of climate change, loss of biodiversity, use of water resources, impairment of nutrient cycles, and changes in land use. These issues clearly highlight the paradox that afflicts food systems: despite the challenge for the coming decades to feed a growing world population that is increasingly concentrated in cities [5,6], the current global food model, dominated by diets strongly impacting on ecosystems and human health, is not able to guarantee this function in a sustainable manner. Furthermore, while 820 million people, equal to 10.9% of the world population, do not have access to sufficient quantities of food has achieved (in 2014, the number of undernourished people was 783 million, equal to 10.7% of the world population), the number of non-transmissible diseases linked to obesity and overweight is growing rapidly [5]. All this happens at a time when, as attested by the last Intergovernmental Panel on Climate Change (IPCC) Special Report on climate change and soil, the availability of food per capita is higher than the average requirement [7,8]. Furthermore, as the 2007/2008 agricultural prices downturn has shown, globalized food systems are fragile, especially in cities, also in light of estimates of urban population growth rates in the coming decades, and put the issue of food security at the center of political agendas. These changes have been so sudden and of such remarkable impact that a new paradigm has been proposed to describe them, the “New Food Equation” [9,10]. The New Food Equationì is based on the evidence that the dynamics concerning the co-evolution between food systems and demographic development are rapidly reshaping the way in which food is produced, processed, distributed, and consumed; impacts the metabolism of a city and its economy; affects public and private spaces [11]; and impacts food consumption patterns and foodscapes.

Numerous factors require a theoretical revision of the relationships that the city weaves with the surrounding territory for the purposes of food production, processing, and distribution. The de-territorialization of food systems [12] has manifested itself in a very decisive manner in recent decades, with evident and dramatic consequences on the ability to manage and govern material (materials raw, processed products, and food waste) and immaterial (knowledge, traditions, and consumer-producer relations) flows related to food [13]. Recently, simultaneously with a growing interest of research and institutions for urban–rural relations, cities have begun to think about how to integrate, connect, and protect green agricultural areas and the social, economic, and environmental functions they provide to the well being of the population [10,14]. Among the approaches to respond to the challenges that involve the transformation of urban food systems, the most widespread today is the City-Region Food System, supported by the Food for the Cities program of FAO in collaboration with the RUAF foundation (Resource Centers on Urban Agriculture and Food Security). It has been defined as the complex network of actors, processes, and relationships affecting the production, transformation, marketing, and consumption of food that exists in a given geographical region that includes a more or less concentrated urban center and its surrounding peri-urban and rural hinterland [15]. The underlying assumption of this approach is that, if policies are developed on a city-region scale, the recognition of the agro-ecosystems’ specificities makes it possible to deal simultaneously with urban issues (food safety and health), agricultural issues (opportunities for local farmers), and environmental issues (risk management). In this context, cities—through diversified approaches and various administrative and functional solutions—are taking on an increasingly central role in trying to manage the food system for better governance of many of the challenges they are facing today. In other words, cities are becoming strategic geographical transition nodes that can exploit the political vacuum generated by the absence of global, coherent, and integrated food policies—national or supranational—to develop more sustainable food systems [16,17]. (When referring to processes towards sustainability of food systems, the authors mean holistic approaches that integrate all aspects of the food system. This includes urban and peri-urban agriculture; strengthening the rural–urban interface to ensure connections between rural supplies and urban contexts; taking into account street food, retailers, food processing and distribution, nutrition, and health; and linkages to water, waste, transport, and energy systems [18].) In this context, urban food policies find their ways and are increasing popularity. Urban food policies have been defined [19] as a concerted action on the part of city government to address food-related challenges. Urban food policies often emerge through significant involvement of civil society and other actors. Most urban food policies consist of targeted actions with specific goals, such as addressing a specific public health or environmental concern (e.g., obesity and food waste). Such actions can pave the way for—and be incorporated into—integrated food policies at a later stage and may also have benefits in other policy areas. In most urban food policies, the reasoning on local food systems is directed towards a physical (and therefore ecological), symbolic, and economic reconnection between the city and the countryside through a series of actions and tools that involve all phases of food chains [19,20]. This implies the recognition of agro-ecosystems and agricultural production not as antithetical activities compared to urban ones, but as integrated phenomena, capable of playing a key role in the development of urban systems. Cities are where the social movements of food re-signification find their place, a privileged lens for observing the transformations of food systems and governing many of the challenges of the coming decades. Indeed, the urban scale has been considered the most appropriate one for a couple of decades [21] to intercept and govern the economic and social dynamics and, above all, the policies, tools, and actions that make food systems more sustainable and compatible with the challenges of the new millennium that the Sustainable Development Goals of the United Nations 2030 Agenda have highlighted. Acknowledging that cities have a strategic role to play in developing sustainable food systems and promoting healthy diets, the Milan Urban Food Policy Pact (MUFPP) was established in 2015. MUFPP represents a global commitment among more two hundred mayors from around the world who consider food as an entry point for the sustainable development of growing cities. It represents the main framework for cities and international stakeholders active in innovative urban food policies for the management and governance of local food systems, and includes more recent experiences as well as cities with relevant good track records in institutionalized food strategies such as Toronto, New York, Bristol, Quito, Belo Horizonte, Montpellier, and many others. This research contributes to the momentum that is creating a fertile ground for the establishment of a food policy for the metropolitan city of Rome. Indeed, as highlighted in the next paragraphs, several political statements and declarations, along with growing pressure for a more sustainable food system from civil society, are strong factors that could lead to an institutionalization of a systemic food strategy for Rome. In this context, analyzing the local food policy group—and its relationships with the administration—contributes to the understanding of its role in the future and its ability to survive political cycles, reflecting upon the pros and cons of the space in which the food policy group is to operate. To better position the case of Rome, an overview of the state of the art of urban food policies in Italy has been provided.

## 2. Research Design

**Figure 1:**
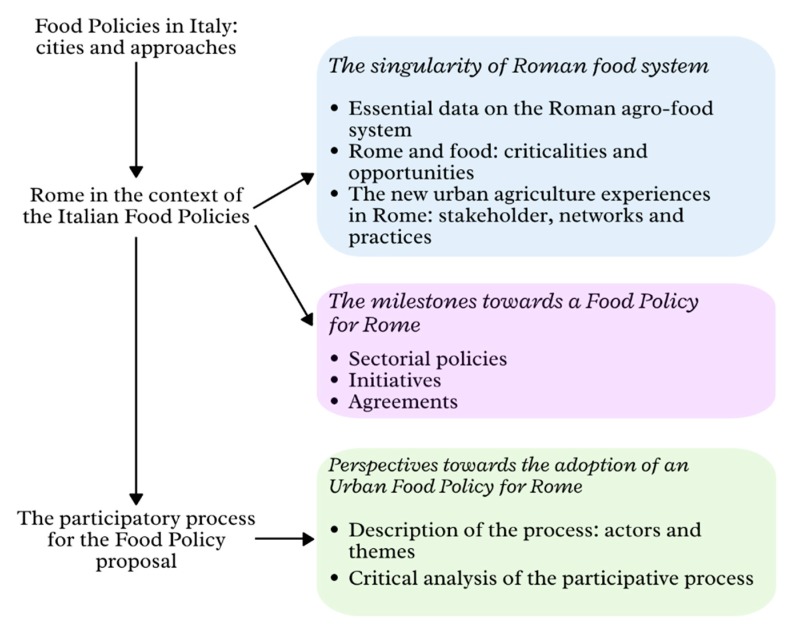
Research design (source: our elaborations).

The relationships between agricultural landscape and city have always determined consumption choices and dietary patterns [22]. The forms of food production give a transformation—physical, but not only—to the natural landscape, through a conscious and systematic anthropic action [23]. Agricultural landscape, diets, and food consumption models are therefore interconnected and mutually dependent. In the Mediterranean diet, these relationships have shaped territories and compositions of foodstuffs that are exchanged and consumed, orienting them towards products such as fruit, vegetables, legumes, seeds, and olive oil, widely recognized as “healthy” for human health [24,25]. However, their effects in terms of environmental sustainability, agricultural systems, and rural landscapes are not unique [26]. This clarification seems necessary to contextualize the agri-food system of Rome, in which the associative movements and the bottom-up participatory path for a food policy proposal have arisen in response to the tendency towards the “westernization” of diets, which, besides being considered risky for human health [27], risk dispersing cultural capital, traditions, and connections with the rural landscape embedded in typical products of the Mediterranean diet. (The Western Pattern Diet (WPD) or Standard American Diet (SAD) is a dietary model generally characterized by high intakes of red meat, processed meat, pre-packaged foods, butter, fried foods, fat-rich dairy products, refined cereal eggs, potatoes, corn (and high fructose corn syrup), and high-sugar drinks.) In the following paragraphs, the process that led a group of local stakeholders (associations, researchers, groups of farmers, and civil society) to formulate a Food Policy proposal for the city is analyzed. The analysis of the process, which mainly investigates the networking and negotiation capacities between various interests, is carried out and related to an overview of the food system in the Roman context, which highlights its territory, agro-food productions, the food distribution model, consumption patterns, and already active experiences, which can play a fundamental role in directing the transition of the local food system towards greater economic, social, and environmental self-sustainability. In Figure 1, the research design has been shown, in order to better orient the reading of the paper.

## 3. Food Policies in Italy: Cities and Approaches

In Italy, the models proposed by the so-called Territorialist School [28,29], ascribable to the approaches attributable to the urban bioregion [30,31], were based on cultural and scientific models such as ecological agriculture, the territorial approach, and the analysis of territorial metabolism and supply chains [32]. In the national territory, the initiatives linked to food policies, in the absence of a national strategy dedicated to food systems, have developed according to specific paths at the local level, around some practices (school canteens, quality food education programs, and short food chains), often based on private corporate and civil society initiatives. Today, 26 Italian cities, out of a total of 207 cities, have signed the MUFPP (as of 2 October 2019), but not all of them are equally committed to more sustainable food systems. The following experiences have been reported to highlight the different entry points towards formalized urban food policies or bottom-up processes in Italy. Our aim is not to compare them, as we are referring to different scales, from metropolis (Milan and Turin) to medium-sized cities (Livorno and Pisa), to small towns (Castel del Giudice and Tollo). Instead, it is useful to provide a synthetic overview of the approaches adopted by Italian municipalities, local groups, or mixed partnerships, in order to understand what role Rome can play in a national view. In Tuscany, the university and researchers have played a fundamental role in the promotion and cultural dissemination of issues related to food planning: the Food Plan of Pisa, adopted by the Province in 2010 [33], represented for some years the national reference point for both participatory processes and relations between the various sectors of the administrations and among administration, citizens and other stakeholders [34]. After Expo 2015 “Feeding the planet, energy for life”, the municipality of Milan implemented its own Food Policy, a strategy based on five priorities: (1) healthy food for everyone; (2) sustainability of food system; (3) food education; (4) fight against food waste; and (5) scientific research on agro-food systems. The Polytechnic University of Turin, in collaboration with the Metropolitan City, recently drafted the Turin Metro Food Atlas, an analysis, representation, and communication initiative of the urban metropolitan food system [35]. A similar initiative was launched in Matera, where, however, the experience of the Food Atlas does not yet involve the local administration but, as stated on the project website, “could become support for the construction of a Food Chart in Matera”. Recently implemented is the Pact for Urban Policies for Food signed by the municipalities of Lucca and Capannori, born following the experiences of the European project ROBUST (Risk and Opportunity management of huge-scale BUSiness communiTy cooperation), a project that analyzes the interrelations between urban and peri-urban areas with respect to territorial planning, culture, production enhancement, and services. Even small municipal entities have realized the potential of local food policies: this is the case, for example, of Castel del Giudice (Molise Region), which through the Food Plan and a virtuous collaboration with the University of Molise has set up a strategy to counter the depopulation of the area and provide opportunities for socio-economic development by leveraging the landscape, agricultural, and environmental characteristics of the place [36]. The Municipality of Tollo (Abruzzo) has instead approved its own Food Policy, with the intention of making the strong wine prevalence a catalyst for triggering integrated processes between local economies, increased biodiversity through crop diversification, improved quality of diet, and increased territorial development. In Bergamo, the Bergamo Green project produced a mapping of the Alternative Food Networks of the province, with the aim of giving visibility to the realities of production, distribution, and consumption of sustainable, local, and organic products, and to favor the path towards the Food Policy of Bergamo. The project Feeding Trento also relied on a mapping of the virtuous experiences of its territory, also making a platform available and establishing a working table managed by a collaboration between the municipality and the University of Trento. Finally, Venice is carrying out an ex-ante analysis, trying to understand how the remarkable tourist flow affects the lagoon food system, in particular the foodscape and the food narratives that are proposed in the city. From a preliminary analysis carried out through an online survey among the members of the Italian Network of Local Food Policies, there are several critical issues related, in the first instance, to the difficulties inherent in the translation of plans, strategies and programs into actual actions on the territory and on the food system. First, there is a strong concentration of policies in the central and northern regions, in which the Food Policy of Milan emerges as the only major city in which the administration has supported the policy thanks to institutional support and a dedicated and participated budget by a foundation banking. An important area of innovation is represented by Tuscany, in which the experiences of Lucca, Pisa, and Livorno suggest the role of the university, and sometimes of individual departments, represents a propelling factor of considerable importance. The themes around which plans and programs are developed mainly include Green Public Procurement in collective catering, often accompanied by attention to the flows—and the consequent agricultural and territorial dynamics—that should fuel the food supply according to the sustainability criteria promoted. Another key theme is food waste, around which the debate is on today but which is often addressed without a systemic view of the problem. In fact, as highlighted in the ISPRA report on food waste [37], the objective of the systemic approach is the protection of joint socio-ecological systems, and not only the efficient use of resources or food security. Finally, among the critical points observed so far in the Italian panorama of food policies, there is a certain propensity of administrations to devote themselves to programs that can be achieved through a modest—or sometimes zero—use of economic and human resources, with the result of a failure or watered-down effectiveness of the measures. This could represent the effect of the lack of a medium- to long-term political vision that could reverberate its own measures on the territorial and land-based structures of the city-region. In this sense, the administrators are probably more worried about communicating and highlighting the efforts within an electoral mandate that is certainly too short compared to a possible transformation and transition of the urban food system. In this context, we are interested in observing how Rome is moving in the panorama of Italian food policies. To this end, an analysis of the Roman foodscape (productions, agricultural models, critical issues, challenges, new economies, and networks) is provided and then the participatory process that led a group of stakeholders, which have been clustered and presented in the next paragraphs, to present some food policy proposals to the administrations is critically investigated. In this context, we refer to the definition of foodscape offered by Mikkelsen [38], which identifies it both as a tool for the study of “food environments” and for the evaluation of potential impacts on food choices and behavior, both as a framework for analyzing how food, places, and people are interconnected and how they interact with each other.

## 4. The Rome Foodscape

### 4.1. Essential Data on the Roman Agro-Food System

The metropolitan area of Rome has a population of about 4.34 million inhabitants for an area of 5352 km^2^. At the municipal level, the Total Agricultural Area (SAT) of Rome is approximately 58,000 ha, or 45.1% of the territory. In the countryside and on the Roman hills, many quality food products are produced and processed (8 Protected Designation of Origin and 7 Protected Geographical Indication), among which stand out products that strongly characterize the territory, the supply chains, and the local cuisine such as the Abbacchio Romano, the Pecorino Romano, and the Ricotta Romana. Historically, sheep and goat farms have represented a fundamental economy for the Roman countryside, substantially determining the landscape, customs, and traditions of the Roman countryside. In Lazio Region, and in particular in the Municipality of Rome, there is a significant presence of livestock holdings in the sheep and goat sector (in 2014, 6500 units in Lazio and 500 units in the Municipality of Rome, according to data of the Livestock Registry established by the Ministry of Health), which, in terms of size, are concentrated in small and medium size classes (number of heads less than 1000) [39]. Sheep milk production includes some P.D.O (Pecorino Romano, Pecorino Toscano, Pecorino di Picinisco, and Ricotta Romana) and numerous Traditional Agri-food Products, into which tangible and intangible values are embedded from the high interaction between natural and cultural capital [40], being the Roman agricultural landscape immersed in a network of pre-existing archaeological sites, monuments, villas, and farmhouses. This framework is enriched by the role that the “Agro Romano” has historically held, in particular for the relevance that some productions have in the local productive fabric. This is the case, for example, of the dairy livestock practiced in the northwest quadrant of the city towards the Via Aurelia, of the horticulture linked to the coastal reclamation plain and the ovine livestock and the agro pastures [41]. In the Municipality of Rome, farmers are mainly dedicated to the production of arable crops (cereals and forage), followed by permanent meadows, pastures, and woody and agricultural crops such as olives and vines.

**Figure 2:**
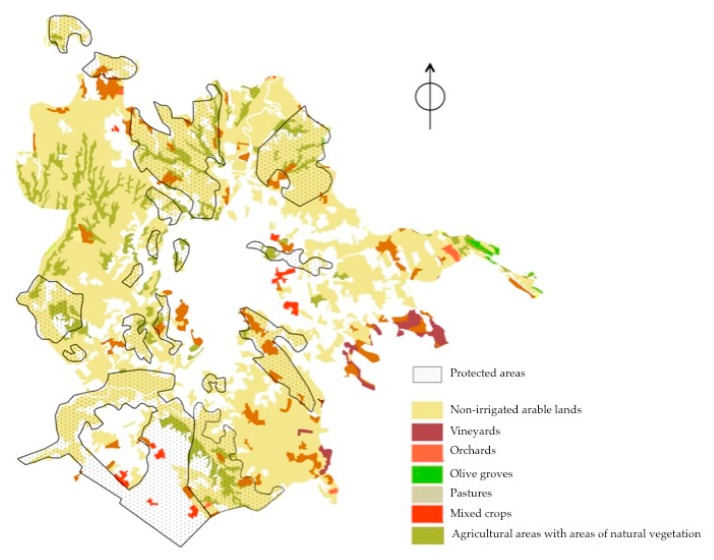
Land uses and protected areas in the Municipality of Rome, scale 1: 100,000 (Source: [42] using data from Corine Land Cover, 2006).

In structural terms, the latest data available on the Roman primary sector show a growth in the number of farms, which mainly concerns small- and very small-scale realities (areas of less than 2 ha). At the provincial level, there has been, over the last decade, a 58% decrease in farms and 8.2% in agricultural areas, while, at the municipal level the figures show opposite signs, and for significant values: +43.8% of farms and +12.1% of Total Agricultural Area, for a total of 57,959 ha (45.1% of the 128,540 ha occupied by the municipality). A fundamental role for the ecosystem services offered to the population is played by the protected natural areas, which constitute an important system of green infrastructures (see Figure 2). These areas are located mainly in the peri-urban area, but also extend to the most central areas and, overall, reach an area of 41,500 ha, equal to 32% of the entire municipal area. According to the current General Master Plan, two-thirds of the municipal territory constitutes the city’s current Ecological Network, an articulated and functional system of areas of naturalistic, agricultural, and recreational importance. It is composed, in fact, by a complex fabric of protected natural areas, urban green areas (historic villas, gardens, tree-lined streets, etc.), blue infrastructures, and agricultural areas. These green areas cover approximately 86,000 ha, equal to 67% of the entire surface of the municipality, offering spaces for the conservation of habitats of particular naturalistic value and a variety of natural environments and ecological niches. (Most of the protected natural areas (14 out of a total of 19 plus the protected marine area) are managed by the regional body “RomaNatura”, as they all fall entirely within the municipal territory.) In this context, agricultural parks play a central role for the green connectivity of Rome and for their high potential in terms of reconstructing the relationship between agriculture and cities, citizens and farmers, and public and private spaces, actively contributing to improving the resilience of the city [42]. (One of the most significant cases is certainly represented by the Casal del Marmo Agricultural Park, in the northwest quadrant of Rome. The area is completely immersed in the urban mosaic and therefore plays a role—real and potential—of providing ecosystem services that are very relevant to the surrounding area. In particular, the analysis of cultural ecosystem services provided by the park was carried out by Davide Pellegrino in a doctoral thesis on landscape and environment entitled “The green infrastructure for landscape governance: the contribution of ecosystem services” [43].) Still referring to the Roman foodscape, it is necessary to mention the abundance of initiatives linked to urban community gardens in the city, historically present and recently reported at the center of the discourses on urban metabolism and social justice. From a survey by the Zappata Romana Urban Architecture Project, which in 2011 compiled a map of shared gardens in Rome, there are 218 recognized associative experiences. A study [44] found 3200 plots of residential gardens (85% of the total), shared gardens, institutional gardens, and informal gardens within the municipality borders. An important project on urban agriculture in Rome is Ru:rban, a European project started in 2019 that aims to exchange good practices between cities and European experiences in the context of urban gardens and agriculture, taking an example from the paradigmatic experience of Rome. Simultaneously with the return of urban agriculture, a phenomenon that has resumed in recent years following a long period of housing development that slowed its development [45,46], is the remarkable development of alternative food networks in Rome: all forms of short supply chains—from farmers’ markets to Solidarity Purchasing Groups (SPGs) and box scheme experiences—have achieved significant success. The numbers of short supply chains in the Roman area present proportions of primary importance in the national context: the municipality of Rome has 33 farmers’ markets and 55 SPGs, while 744 out of 2,656 farms of the area sell direct (with a 40% increase in the last census interval 2000–2010).

### 4.2. Rome and Food: Criticalities and Opportunities

It has been highlighted that the foodscape of the Roman area is particularly rich in experiences, productions, and practices related to innovation in the agro-food sector. Rome and the surrounding countryside represent a considerable basin for the production of food and agricultural services, a characteristic that is not common if one looks at the territorial composition of many western metropolises. However, practices and experiences appear disconnected, poorly interconnected and, above all, exposed to the risks of an agro-industrial market that reduces the space for small traders. Among the critical issues that undermine the value of the Roman agro-food system, it is necessary to mention the land use changes in place in the Municipality of Rome. The 2018 report on the Land use in Rome [47] shows that 23.5% (30,241 ha) of the territory is covered by artificial surfaces. It can be noted that, in the face of an almost zero increase in the population, the consumption of soil is growing. In fact, even in sparsely populated neighborhoods and in demographic decline areas, the impermeable surfaces are stably increasing over time. The same report indicates that an area of 6332 ha is characterized by the maximum hydraulic danger and 14,588 residents are at risk. To the extent that land use change is a theme that is shared and worries most of the metropolis, it is to be noted that, in the Roman context, the disruption of the relationship between farmers and consumers due to the processes of standardization and globalization of the supply chains in recent years has produced a series of critical issues that reverberate on the markets and, therefore, on the purchasing methods and consumption patterns of Romans. Indeed, although the local markets represent a source of food supply for Romans from the post-war period onwards, they are going through a moment of crisis due to confused and inefficient regulation and the competition of the Organized Large-Scale Distribution system, where about 70% of purchases take place at the national level. According to the latest census of local markets, carried out in 2015 by the Economic Development Department, there are 127 food markets in the capital, with a total of over 2500 sellers and 5000 stations. Nevertheless, merchants often mediate access to markets, with only one hundred farmers selling their products directly. Over the years, these markets have experienced an almost constant decline, resulting in many semi-abandoned and customer-reduced facilities [48]. Local markets have the fundamental role as a hub for the distribution of products grown in Rome and Lazio. However, the relationship between city and countryside is influenced substantially by the land composition of the territory, the presence of abandoned lands—whether they are private property or state property—and the contractual relations between farm managers and owners. However, given the wide availability of agricultural land, the supply of regulatory instruments for access to them could be developed and integrated into a food policy in which the settlement of new—possibly young—farmers is one of the cornerstones for the revitalisation of a re-localized agro-food sector. In 2014, the Municipality of Rome promoted the program “Rome, city to be cultivated”. The tender for the assignment of land and rural buildings owned by Rome was aimed at protecting and restoring the Roman agro-production, by developing multi-functional farms, rewarding the orientation of competitors towards organic farming and multi-functionality. Nevertheless, the works for the renovation of rural buildings are currently at a standstill and the effective incidence of the assignable lands on the total of the available lands is minimal: only a quarter of the roughly 26,000 ha of public lands is the object of assignment and multi-functional agricultural projects have been approved on only 95 ha. In terms of re-territorialization of production and the triggering of virtuous processes in support of local agriculture and nutritious and sustainable diets, the Green Public Procurement also intervenes substantially. Indeed, in Italy, all contracting stations—including municipalities—are obliged to respect the rules established by the Minimum Environmental Criteria, in accordance with a law issued by the Ministry of the Environment. Rome offers school catering services daily to around 144,000 pupils. In the Municipality of Rome, between September 2007 and June 2012, approximately 67.2 million meals were distributed in school canteens, and between January 2013 and June 2017 approximately 71.4 million meals were distributed, for a total of about 138.6 million meals in ten years. These data tell us that, by working on the tender specifications (the value of the last call for tenders is more than 350 million euros over three years) to follow short supply chain criteria, sustainability, and seasonality, important effects could be obtained on the fabric urban, peri-urban, and rural economic and educational levels. Broadening the analysis of the Roman foodscape towards the world of food distribution, in Rome, supermarkets show growing numbers. The 2016 data of the Ministry of Economic Development identify 30 hypermarkets (+61% from 2013 to 2017) and 520 supermarkets in the province of Rome (329 in the municipality), growing in the last five years. With reference to the municipality, there are over 500 supermarkets, with a total sales area of over 290,000 square meters and 7000 employees, which are associated with 194 minimarkets, which in turn cover an area of approximately 36,000 square meters and have almost 900 employees. Rome is first in Italy for shops specializing in organic food (118 out of 1437). At the same time, Rome is one of the cities in which the phenomenon of minimarkets managed by Egyptian or Bengali personnel has strongly grown in recent years. The ease with which a license can be obtained, combined with the fact that retail trade and self-entrepreneurship are almost obligatory choices for social groups forgotten by employment policies, has led these minorities to taking over hundreds of small shops from Italian merchants. Usually, they remain open until late at night, including Sunday, and offer various kinds of products, including low-cost fruit and vegetables. Finally, it is necessary to mention the distortions linked to food waste, which in Italy are worth over 15 billion euros (almost 1% of GDP). Considering that the estimates on the quantity of food thrown out in Italy by wholesale and organized distribution markets amount to about 400,000 tons of food—40% of which is made up of fruit and vegetables—in the metropolitan area of Rome, there could potentially be 29,000 tons of recoverable and redistributable food a year. In Rome, numerous experiences were born with the aim of guaranteeing a second life for food that is not consumed or purchased. The law of 19 August 2016, No. 166 (the so-called Gadda law) encourages and facilitates the distribution and recovery of foodstuffs to poor people and allows a reduction in the waste tax. In 2017, Rome approved a plan for the reduction and management of post-consumer materials in Rome Capital 2017–2021, which aims to bring Rome closer to a circular economy with zero waste. The goal is to reduce the annual waste production by 200,000 tons by 2021, increase separate waste collection from 44% to 70%, and create new recycling and composting plants. The Food Sharing project is part of the plan, which provides for the systematic withdrawal of fresh food from shops, supermarkets, and local markets to subsequently redistribute them to non-profit organizations dealing with people in difficulty and to the Bioparco Foundation, which allocates unsold foodstuffs to the hosted animals. Other virtuous experiences, such as ReFoodGees, assume principles of circular and solidarity economy to activate food recovery and distribution actions, with the aim of creating spaces for socialization, combating waste, and social exclusion and discrimination.

### 4.3. The New Urban Agriculture Experiences in Rome: Stakeholder, Networks and Practices

The panorama described thus far reveals, on the one hand, the centrality of agriculture for the Roman territorial system, the liveliness of some movements (urban gardens and alternative food networks), and the relevance of the interaction between cultural and natural capital for Rome, and, on the other hand, the critical issues related to urban development that create significant pressures on agricultural land and the associated economies and a lack of integrated and long-term regulation of spaces, uses, and markets. More than forty years after the movements that led to the creation of numerous agricultural cooperatives linked to the recovery of uncultivated land owned by the state, there are still today several experiences that invest in innovation, diversification, and the relationship with the local community. However, the problems linked to the development of low-density housing settlements, the abandonment of large areas that were previously semi-natural, and the consequent loss of environmental functions are putting at risk the ability to provide citizens of urban areas with those fundamental services for the good quality of life in the city. As mentioned above, Rome boasts one of the largest non-urbanized areas in Europe. This aspect makes it particularly suitable for hosting urban and peri-urban forms of agriculture. These experiences are mostly of a voluntary nature and pursue social and community aims. However, there are an increasing number of multifunctional urban agriculture initiatives for commercial and entrepreneurial purposes, which produce private incomes but reverberate the positive externalities on the community. These farms adopt strategies to adapt the management and commercial organization to seize the opportunities of Rome’s large and dynamic urban market. The particular vivacity of the bottom-up movements that distinguish Rome from the post-war period onwards led “Agro Romano” to be the fertile ground for the development of pioneering experiences of social agriculture at the national level starting from the end of the 1970s, concomitantly with the bottom-up recovery movements of abandoned land. This aspect has made the Roman territory a laboratory of social innovation practices in which social agriculture has always played a central role. In the municipality of Rome, there are today 32 farms conducting social agriculture (Capodarco cooperative is among the most famous one, also at national and European level), to which another 20 must be added in the metropolitan area. There are more than 1740 end users at the regional level. With regard to collective processes, an important process of legitimizing and regulating urban gardens in the city of Rome is now carried out by the OrtiInComune network. Animated by passionate horticulturists and citizens, through a constructive and constant dialogue with the Rome administration and representatives of the various municipalities, the network cooperates in facilitating the political processes that govern the management of urban green spaces, including urban gardens. In addition, the network is active in awareness-raising campaigns, training days, and cultural activities, substantially contributing to the public debate in the city linked to the themes of public green areas and food. In this context, the experiences of solidarity-based economy, which in Rome are channeled into the Network of Social and Solidarity Economics (Rete di Economia Sociale e Solidale (RESS)) are growing their relevance. (The Social and Solidarity Economics Networks are concrete and practical alternatives to create an economy and a society oriented to the well-being of all. The idea behind social and solidarity economy networks is the concept of reciprocity, or the exchange of favors directly or indirectly, between different subjects of society, for “duties” of solidarity.) It involves the different Solidarity Purchasing Groups (SPGs) of the territory, consumers, producers, and suppliers; organizations and associations that deal with solidarity economy; and individual citizens. RESS periodically organizes meetings and activities, both to talk about different models of small organized solidarity distribution and to exchange information and knowledge on the topics of food sustainability and distribution of added value along the food chains. Another experience that involves different SPGs is RESS Ciociaria, which brings together different purchasing groups in Lazio to promote sustainable and conscious consumption and production.

## 5. The Participatory Process for the Food Policy Proposal

### 5.1. Description of the Process: Actors and Themes

**Figure 3:**
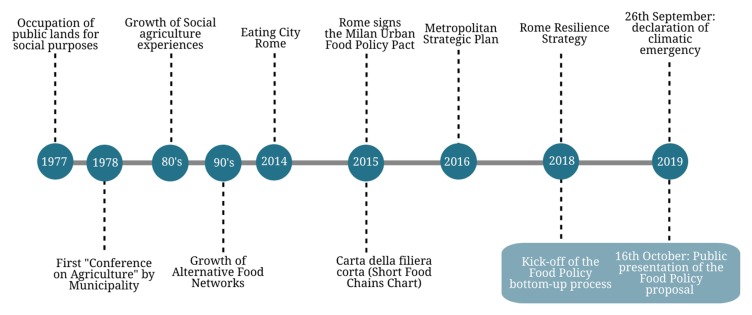
Milestones towards a Food Policy for Rome. Source: our elaborations.

In light of the social liveliness of the Roman foodscape, in October 2018, a bottom-up participatory process started, aiming at formulating a proposal for an urban food policy for the city of Rome to be presented to the city and local institutions. (It has to be specified that the authors participated as moderators and facilitators of the meetings of the local food policy group and that the following analysis is based on the minutes of the meetings, the comments reported by email via the dedicated account, and the feedback provided by the participants.) The work started from the awareness of the lack of integration between the experiences seen above both horizontally—that is among them—and vertically—that is with respect to the various governance levels that intervene in food systems. Rome is one of the 26 Italian cities that has signed the MUFPP Pact; however, the administration has not yet committed itself to defining a food strategy, except for the sectorial initiatives mentioned in the present work and the adhesion to international (100 Resilient Cities) or European (Urbact and Ru:rban) projects. The presence of important international institutions operating in the food and rural development sector (FAO, World Food Program, IFAD etc.), make fertile ground for the opening of a debate on a Food Policy in Rome. Furthermore, Rome has already adopted guidelines in favor of urban resilience by joining the international 100 Resilient Cities program, which provides indications for supporting a more sustainable agro-food system. Finally, with the approval of the climate emergency motion, the municipality has undertaken to “pursue a Food Policy aimed at improving the interconnection between production and consumption, with a view to environmental and economic sustainability”. Finally, in the Metropolitan Strategic Plan, among the 10 programmatic macro-objectives, there are the enhancement of the links between city and countryside and the promotion of Natural and Cultural Capital [49] (see Figure 3).

In specifying that the process is still in progress, we can recognize that the objective of the work group was threefold: (i) to show the municipal and metropolitan administration that the Roman agro-food system has enormous potential and many threats and critical points that should be considered by policy-makers to face the different challenges that the city faces; (ii) to stimulate the participation of civil society in the debate on the food system, increasing awareness of the potential impact of a food policy, and increasing the social capital and trust between operators and economic actors; and (iii) to provide and suggest tools, actions, and concrete measures for the implementation of a systemic food strategy for the city of Rome.

Thus far, the participatory process has involved many stakeholders, stimulated by a group of academics from the University of Molise and Roma Tre University, independent researchers, journalists, and activists in the world of associations. Today, the group counts 116 participants, including representatives of: associative movements linked to urban gardens, multi-functional farms, foundations and organizations active on environmental and food issues, professional agricultural organizations, research institutes, associations and social and solidarity economy networks, private operators active in the various phases of the food chain, and interested citizens. The first outcome produced by the promoters has been a report showing why Rome needs a food policy, starting from an analysis of the agro-food system and then formulating a list of ten proposals (the report (in Italian) can be downloaded at this link: https://www.politichelocalicibo.it/wp-content/uploads/2019/10/Una-Food-Policy-per-Roma.pdf). The participatory process has continued through several meetings and the request for amendments and comments to the document by the stakeholders involved, a path that led to the signing of a list of ten policy objectives to be presented to the administration. In the final part of the document, in fact, in light of the evidence that emerged comparing the state of the art, the mapping of innovative practices, and the policies currently in place, it is stated that it is necessary to implement actions aimed at strengthening and supporting small- and medium-sized farms and companies that populate the Roman primary sector along all the steps of the supply chain, from production to marketing, up to the post-consumer phases. It is also stated that, to ensure healthy nutrition and access to quality food for all citizens, while at the same time taking care of the protection of natural resources, it is necessary to support and enhance the good practices of food chains, “having in mind to strengthen the economic and social ties with the rural areas close to the metropolis”. Finally, it is reported that a Food Policy should also be aimed at encouraging generational turnover in agriculture, as well as food education and waste reduction, through both prevention and recovery and redistribution of surpluses. (The ten food policy objectives that the document “offers” to the administration can be traced back to the following topics: (1) access to resources (land, water and agro-biodiversity); (2) sustainable agriculture and biodiversity (support for organic farming and agro-ecology); (3) short supply chains and local markets (including local markets); (4) urban–rural relations (integration between supply chain phases; Green Public Procurement); (5) food and territory (territorial labeling, traceability of the supply chain); (6) waste and redistribution (support for recovery and redistribution of surpluses); (7) promotion of multi-functionality; (8) awareness of citizens (food and environmental education plan); (9) landscape (curbing land consumption and other phenomena of land degradation); and (10) planning of resilience (green infrastructures and quantification of services provided by the agro-silvo-pastoral system to the community).) As mentioned above, the participatory process was fueled above all by a series of meetings in which the priorities for the Roman agro-food system were discussed. This last phase led to the organization of five thematic working groups (access to resources; collective/school catering and Green Public Procurement (GPP); agriculture and workers’ rights; distribution and consumption; and solidarity economy, food poverty, waste, and redistribution) and a cross-sectional working group on communication.

### 5.2. Critical Analysis of the Participative Process

To analyze the participatory process from a conceptual point of view, the stakeholders have been grouped into six clusters: research/university; cooperatives and farmers; urban gardeners; Associations (agriculture/food/environment); civil society; networks for local sustainable development. The clusters have been created to detect the topics and themes that have been raised by them during the meeting of the local food policy group. They have been built through a qualitative and discretional work of allocation performed by the authors. Their composition is described in Table 1.

**Figure 4:**
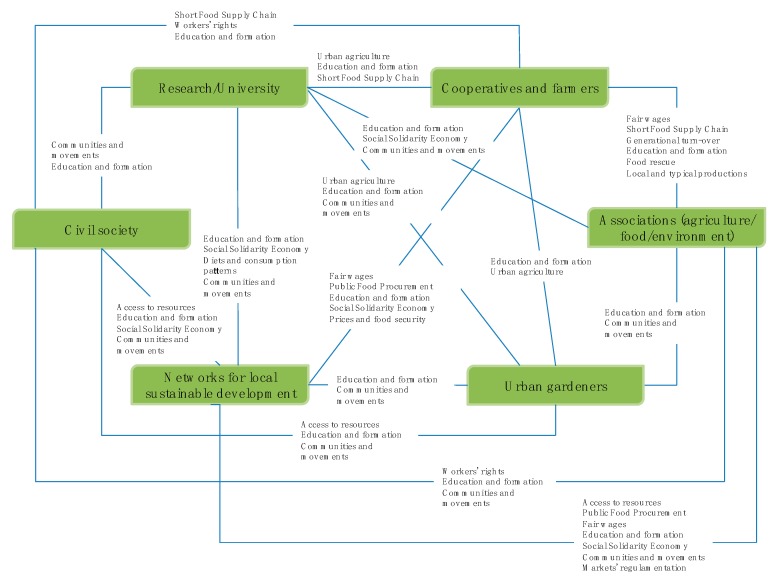
Conceptual map of the actors and themes. The themes common to each pair of clusters are in order of relevance from top to bottom (Source: own elaboration).

The direct participation of the authors in the meetings, the minutes of the meetings and the exchanges of views, and the debate that took place in the specifically created mailing list made it possible to outline the main topics of debate and the positions of the participants. The conceptual graph below (Figure 4) shows which themes triggered the interactions between the six clusters.

It can be observed how some themes have been dealt with in a more widespread and in-depth manner during the meetings. These are issues related to education and training in the agro-food sector, access to resources (mainly the lands) and social and solidarity-based economies, and the associated movements and communities. It is evident that the very composition of the stakeholder group, in which the presence of associations and networks involved in food democracy issues is very strong, has influenced and addressed the debate. Some topics, such as distribution and logistics, have been dealt with to a much lesser extent, due to the absence of operators able to direct the dialogue towards understanding the difficulties and opportunities relating to the modalities with which the food arrives in the city. It can be affirmed that requests and positions relating to alternative forms of marketing of agro-food products are more represented, as many of the actors are directly or indirectly involved in supporting short supply chains. Despite the sustainability of these practices compared to the large-scale retail trade has been demonstrated by the analysis of cases of Italian Alternative Food Networks [50], the participation of representatives of the conventional supply chains in the participatory process would allow grasping the diversity of approaches and needs of a sector that still conveys most of the food that arrives in the city [51] and that must be taken into account in the definition of a systemic food strategy on an urban scale.

The analysis made it possible to identify areas of common interest among the various stakeholder clusters. The results can be viewed in Table 2, which for each pair of clusters identifies projects, possible actions, and collaborations that have arisen during the meetings. At the same time, they represent possible fields of practical application of a food policy for the city of Rome, which could leverage these elements as catalysts for a systemic strategy capable of orienting the entire agro-food system towards an ecological agro-food transition.

## 6. Conclusions

In this contribution, the authors offer a critical analysis of the ongoing process, highlighting the salient passages, strengths, critical points, and above all the future prospects in terms of taking charge of the food question by a city such as Rome, which shows ample room for improvement on the front of integrated and coordinated public policies in support of a more sustainable food system. The variety of practices that animates the Roman context affirms that the city has already started reconsidering food towards more innovative production, distribution, and consumption models, in line with the principles of sustainability and resilience. In fact, three factors intervene to support this hypothesis: (i) Rome is characterized by many experiences related to sustainable food. However, at the political level there is as yet no defined strategic vision and direction, with the risk that such initiatives lose the ability to accompany the transition to sustainable food systems. (ii) There is an interesting mix of bottom-up initiatives and sector institutional tools/incentives/actions. However, these two worlds are often unrelated and lack connections, spaces for debate, and political coordination. (iii) There is an agricultural mosaic of considerable value, but it is not adequately supported. The most pressing challenges include the fragmentation of the agricultural landscape and the fragility of urban markets. Some considerations emerge from the critical analysis of the ongoing participatory process, which also reflect some of the opinions expressed by the stakeholder group during the meetings that began in October 2018. Today, an effort is needed to try to hold together two models, that of alternative production and marketing forms [52] and that of conventional practices related to large-scale distribution, and work towards rationalization—which does not mean trivialization or simplification, but rather the recognition of diversity—of the Roman food system. The system must necessarily mediate—in a rational and critical way—between different models, having in mind that each of them brings different benefits to the community and the territory. This seems even more necessary to avoid the risk that sustainable food production and consumption practices do not remain as niches, but can be compared with the market and with more conventional models. The institutionalization of an urban food policy is a fundamental step to harmonize the multiple instances that come from a fragmented agro-food system and at the mercy of increasingly fluid and financial markets. As recognized in research on the Local Food Policy Groups in Bristol and London [53], local administration support helps to integrate sustainable food-related objectives within policy areas that are under the mayor’s direct control. At the same time, mayoral support provides status and legitimacy to food issues. Nevertheless, institutionalizing a Food Council can also come with some downsides: the main risk is being too closely associated with one political personality. Political differences within local government structures can also be an issue. In London, for instance, boroughs (local governments within the city) that were led by a different political party to that of the mayor may have been less willing to cooperate over food. The management of the food issue should be a duty on the part of the Roman administration, which, having signed the MUFPP, has thus far only put in place some sporadic institutional initiatives for the sustainability of the food system, in most cases guided and financed by participation in European projects. As stated in a note from the FAO Working Group “Strengthening Urban Rural Linkages Through City Region Food Systems”, “[...] initiatives rooted in multi-stakeholder participation will be more effective in reaching their objectives, more transparent/accountable in the use of resources, and to ensure longer-term sustainability beyond temporary government administrations. The processes of dialogue and discovery of common interests across urban and rural landscapes will have to be institutionalized in order to last, and to institutionalize governance structures and mechanism will need policy support, resources, and capacity building”. The local group of actors who are determining their overall strategy must reflect upon the pros and cons of the space in which the food policy group is to operate. What are the benefits of their institutional home? What are the drawbacks? How can they work around these? How can food policy councils take their ability to survive political cycles very seriously? Being aware that the process for a food policy in Rome is at its early stage, the reported analysis suggests some reflections. On the one hand stands the importance of the role of movements from the bottom—above all for their ability to take action and work regardless of the duration of the electoral mandates of the administrations, too short compared to an effective transition of the food systems. On the other hand is the need to institutionalize food policies to provide adequate support in terms of resources, political support, and the spread of consent.

## Figures and Tables

**Table 1:** Description of the clusters composing the local food policy group in Rome (Source: own elaboration).

Cluster	Number	Percent	Description
Research/University	31	26.7%	Representatives of Universities, center of research on agricultural and food economics, independent researchers, representatives of research departments within foundations, companies and associations which link academic research with local development strategies or international exchange programs.
Cooperatives and farmers	12	10.3%	Farmers (mostly running multifunctional farms), representatives of social agriculture experiences, representatives of agricultural cooperatives and one agronomist.
Urban gardeners	7	6.0%	Representatives of urban gardens associations, public local agencies for the promotion of urban gardens in Rome, representatives of European projects on urban gardens, a private company that organize events for participatory processes aimed at improving urban gardens and that performed a mapping of urban gardens in Rome
Associations (agriculture/food/environment)	36	31.0%	Local associations and Roman seats of national and international associations. The associations are active in the fields of the protection of the environment, on the farmers’ rights, on the fair access to agricultural resources, on the promotion of high-quality and fair agro-food products, on food rescue and waste prevention initiatives, on the promotion of agroecology and organic agriculture, on the defense of specific aspects (bees and pollination, organic agriculture in mountain areas), on the support to the achievement of Sustainable Development Goals.
Civil society	20	17.2%	Citizens interested in participating the local group, networks of students, associations active in topics marginally related to food systems (human rights), independent journalist.
Networks for local sustainable development	10	8.6%	Networks for Solidarity and Social Economies, public and private local agencies involved in local development programs, foundations working of sustainable development.
	116	100%	

**Table 2:** Thematic areas and common projects developed during the participatory process (Source: own elaboration).

	Research/University	Ag Cooperatives and Farmers	Urban Gardeners	Associations (Agriculture/Food/Environment)	Civil Society	Networks for Local Sustainable Development
**Research/University**						
**Agricultural Cooperatives and Farmers**	Search for urban and peri-urban agriculture solutions as a response to pressures (access to resources) and opportunities in the city (markets): multi-functional farms short supply chains, etc.					
**Urban gardeners**	Collaboration in the quantification of ecosystem services provided by urban gardens (particularly, the cultural ES). Training organization and dissemination activities	Exchange of practices and agronomic technical information. Collaboration in the organization of events and training days.				
**Associations (agriculture; food; environment)**	Collaboration in structuring the forum on food policy and in identifying and dealing with work areas.	Cooperation in the search for solutions to favor generational turnover and promote sustainable and multi-functional forms of agriculture.	Organization of training days. Studies on the replicability and trans-scalarity of urban gardens. Participation in financing projects.			
**Civil Society**	Identification of stakeholders from areas such as rights, social inequalities, and civic networks. Collaboration in the promotion of the forum.	Search for short supply chain solutions to meet the demand for quality food and products while respecting the environment and workers’ rights.	Exchange of visions about the goals of urban gardens and their future development, as well as in relation to urban social issues such as urban expansion and social inequalities.	Development of proposals and possible solutions regarding the issues of access to quality food (food security) and the development of a food community at the urban level.		
**Networks for local sustainable development**	Public food procurement as a set of tools for the re-territorialization of agriculture and the shift towards healthy and sustainable diets	Activation of networks of producers and consumers through innovative economic forms (CSA) and recognition of fair wages	Collaboration on the issues of access to resources (for example water) and how to increase citizen participation (stakeholder engagement).	Development of thematic projects such as the Public Food Procurement, the social and solidarity economy, access to resources (primarily land), and training in agriculture.	Development of reasonings and proposals concerning the need to train the population to adopt healthier and more balanced diets, working through the Public Food Procurement	
